# Advances in Breast Cancer Research: Immunological, Pathological, and Pharmacological Perspectives for Improving Patient Outcomes

**DOI:** 10.3390/ijms27041902

**Published:** 2026-02-16

**Authors:** Susanne Crocamo, Everton Cruz dos Santos, Eliana Abdelhay

**Affiliations:** Instituto Nacional de Cancer, Rio de Janeiro 20560120, Brazil; crocamo07@gmail.com (S.C.); evertoncruzsantos@gmail.com (E.C.d.S.)

**Keywords:** breast cancer, precision medicine, immunotherapy, biomarkers, checkpoint inhibition, Tumor-Infiltrating Lymphocytes, CAR T-cell, vaccines, bispecific antibodies, Antibody–drug conjugates, digital pathology

## Abstract

Breast cancer remains the most frequently diagnosed malignancy worldwide. Over the past decade, advances in molecular biology have expanded beyond tumor-intrinsic features to encompass the immune microenvironment and patient-specific pharmacogenomic profiles, profoundly reshaping diagnostic, prognostic, and therapeutic paradigms in breast oncology. Owing to rapid technological progress and an expanding therapeutic armamentarium, periodic synthesis of both foundational principles and emerging evidence remains essential for the critical interpretation of ongoing advances. This review provides a comprehensive overview of the contemporary global landscape of breast cancer, integrating developments in diagnosis, risk stratification, and therapeutic innovation. We examine the emerging technologies that are redefining tumor characterization, including digital pathology, artificial intelligence-assisted morphological and molecular analyses, and advanced molecular profiling approaches that increasingly inform prognostic and predictive assessment. We further discuss how these diagnostic frameworks are translating into therapeutic advances, with emphasis on immunotherapy, antibody-drug conjugates, mutation-directed targeted agents, therapeutic vaccines, and bispecific antibodies. Collectively, these developments highlight key translational research priorities, support evidence-based clinical decision-making, and explicitly acknowledge disparities in access and implementation between high-income settings and low- and middle-income countries (LMICs).

## 1. Introduction

The intrinsic biological heterogeneity of breast cancer has presented challenges in oncologic practice for decades. This heterogeneity is characterized by diverse histologic types, heterogeneous molecular profiles, variable proliferation rates, multiple genomic alterations, and distinct clinical phenotypes, thereby complicating disease management [1].

In this context, and despite inherent limitations in sensitivity, specificity, and capacity to fully individualize clinical decision-making based on tumor biology [2], conventional diagnostic and therapeutic standardization remain indispensable for establishing tumor origin, defining disease classification, and guiding therapeutic selection [3]. Although such approaches do not capture the full molecular and immunological complexity of breast cancer, their systematic implementation is imperative and particularly critical in low-income settings, where breast cancer mortality remains disproportionately high [4]. In these regions, limited access to pathology infrastructure frequently leads to delayed, incomplete, or suboptimal diagnoses, thereby directly compromising timely treatment initiation and clinical outcomes.

Accordingly, successive waves of technological innovation have expanded the diagnostic lens beyond morphology and single-protein markers, including immunohistochemistry (IHC)-based profiling of estrogen and progesterone receptors, human epidermal growth factor Receptor 2 (HER2), and Ki67 enabled practical subtype assignment (luminal A, luminal B, HER2-positive, and triple-negative (TBNC) [5], while the advent of genomics, transcriptomics, proteomics, and metabolomics have revealed additional layers of variability with direct prognostic and therapeutic implications [6,7,8].

The molecular characterization of breast cancer has evolved substantially over the past two decades, driven by high-precision technologies that extend beyond conventional histopathological classification. Transcriptomic profiling, initially based on DNA microarrays, has enabled the identification of the gene expression patterns associated with distinct tumor biology and treatment response, leading to the definition of intrinsic subtypes, luminal A, luminal B, HER2-enriched, and basal-like, with clear prognostic and predictive relevance [9].

Advances in next-generation sequencing (NGS) panels have expanded the detection of these alterations, enabling detailed molecular mapping in tumor tissue and biological fluids [10,11,12].

**Figure 1:**
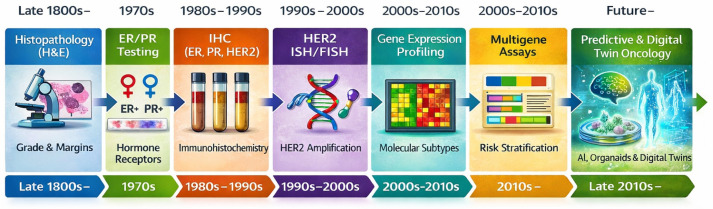
Timeline of pathology-based diagnostics in breast cancer. Schematic timeline summarizing the evolution of pathology-driven diagnostic approaches, from conventional histopathology (H&E) and hormone receptor testing to routine immunohistochemistry, *HER2* confirmation by ISH/FISH, gene-expression profiling and multigene assays, ctDNA liquid biopsy, and digital/computational pathology. Created in OpenAI (2026) ChatGPT/DALL-E3 (version 5.2) on 10 February 2026, https://openai.com/.

As summarized in Figure 1, this evolution reflects a shift from morphology-centered pathology toward integrated molecular and digital diagnostics, including confirmatory in situ hybridization for actionable targets, multigene expression signatures, and NGS enabled tumor genomics for refined prognostic/predictive stratification, and more recent implementation of circulating tumor DNA (ctDNA)-based liquid biopsy and computational pathology to support the longitudinal monitoring of clonal dynamics and treatment resistance. Against this backdrop, precision oncology increasingly relies on the convergence of molecular pathology, tumor immunology, and pharmacologic innovation to deliver biomarker-driven strategies that combine targeted therapy, immunotherapy, and predictive response models, thereby advancing individualized care across the breast cancer continuum [13].

Breast cancer is a heterogeneous disease driven by multi-omic alterations (genomic instability, transcriptomic reprogramming, epigenetic dysregulation, and proteomic/metabolomic heterogeneity) within a complex tumor microenvironment (TME) enriched by immune evasion and stromal interactions. Big data analytics (BDA) integrates multi-layered datasets (genomic (WGS/WES, RNA-seq), clinical (electronic health registries), imaging (radiomics), and environmental/epidemiological) to uncover nonlinear associations, such as subtype-specific driver variants, immune signatures, and treatment response predictors. BDA enables identification of actionable vulnerabilities, facilitating precision oncology by linking molecular phenotypes to therapeutic susceptibility and resistance mechanisms [14,15,16]. These advances were only possible due the global initiatives generating multi-layered datasets through public databases, e.g., ONCOPOOL-A, which includes patient characteristics, tumor characteristics, pathology, therapies and outcomes [17], the Breast Cancer Gene Expression Miner (bc-GenExMiner) database, which includes gene expression data, microarray and RNA sequencing, tumor grade, size, stage, patient survival, and treatment history [18], the Breast Cancer Data Base, Sweden 2.0 (BCBaSe 2.0), which includes demographic information, tumor characteristics, treatment regimens, and outcomes [19], the Cancer Genome Atlas (TCGA) database, which includes genomic, transcriptomic, epigenomic, and proteomic data, as well as clinical and demographic information for cancer patients [20], and the Molecular Taxonomy of Breast Cancer International Consortium (METABRIC), which includes gene expression, copy number, gene mutation, and clinical data [21].

Big data analytics (BDA) is a cornerstone of precision oncology in BC, integrating multi-omic, clinical, and environmental datasets to uncover subtype-specific drivers, immune phenotypes, and therapeutic possibilities. By enabling advanced risk stratification (polygenic risk score (PRS), radiomics), early detection (liquid biopsy), and treatment tailoring (multi-omics predictive models), BDA advances beyond traditional biomarkers toward personalized prevention and therapy, particularly in high-risk or refractory subtypes [22].

**Figure 2:**
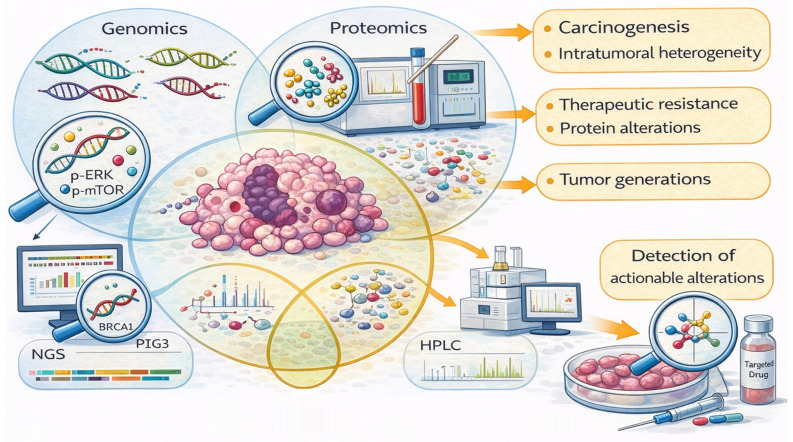
Conceptual schematic illustrating the integration of genomics, proteomics, and metabolomics to characterize carcinogenesis, intratumoral heterogeneity, and therapeutic resistance. Multi-omics profiling enables the detection of recurrent somatic mutations, deletions, gene amplifications, and proteomic/metabolic alterations associated with distinct clinical outcomes, thereby supporting biomarker discovery and the development of targeted therapeutic strategies. Abbreviations: NGS, next-generation sequencing; HPLC, high-performance liquid chromatography; p-ERK, phosphorylated extracellular signal-regulated kinase; p-mTOR, phosphorylated mechanistic target of rapamycin Kinase; *BRCA1*, BRCA1 DNA repair associated; *PIG3*, Tumor Protein P53 Inducible Protein 3. Created in OpenAI (2026) ChatGPT/DALL-E3 (version 5.2) on 10 February 2026, https://openai.com/.

The integration of multi-omic data has improved the understanding of carcinogenesis, intratumoral heterogeneity, and therapeutic resistance, allowing for the detection of recurrent somatic mutations, deletions, gene amplifications, and proteomic/metabolic alterations associated with different clinical outcomes, as well as enabling the development of targeted drugs [11,23] (Figure 2). In this context, the identification of somatic mutations (*PIK3CA*, *TP53*, *ESR1*), germline alterations (*BRCA1/2*), and other genomic events have made the development of targeted drugs and biomarker-guided interventions adapted to specific molecular signatures possible [11].

The development and validation of multigene platforms in early-stage hormone receptor-positive/HER2-negative disease, such as Oncotype DX (21 genes), MammaPrint (70 genes), Prosigna (PAM50), and EndoPredictClin (12 genes combined with two clinical risk factors: nodal status and tumor size), represented a major milestone in treatment individualization by estimating chemotherapy benefit and supporting decisions regarding extended endocrine therapy while reducing unnecessary toxicity and cost [24,25,26,27].

**Figure 3:**
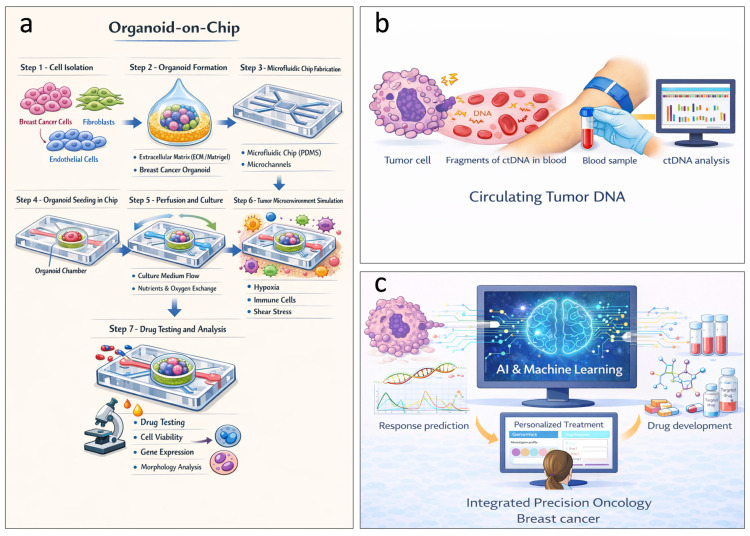
Translational platforms for precision oncology: liquid biopsy, organoid functional testing, and AI-enabled modeling. (**a**) Organoid-on-a-chip platform for functional modeling and drug testing. Microfluidic culture of a 3D tumor organoid under controlled perfusion enables dynamic exposure to therapies and functional assessment of treatment sensitivity. (**b**) Circulating tumor DNA (ctDNA): liquid biopsy workflow from tumor shedding to molecular profiling. Tumor-derived DNA fragments enter the bloodstream and are analyzed (e.g., targeted sequencing) to detect and track tumor-specific alterations for treatment selection and response monitoring for ctDNA blood collection and downstream analysis. (**c**) AI and machine learning in oncology: therapy response prediction and accelerated drug development. AI/ML models leverage biomedical data to improve response prediction and streamline drug development through faster target identification and compound prioritization. Abbreviations: ctDNA, circulating tumor DNA; AI, artificial intelligence; ML, machine learning; ECM, extracellular matrix; PDMS, polydimethylsiloxane. Created in OpenAI (2026) ChatGPT/DALL-E3 (version 5.2) on 10 February 2026, https://openai.com/.

More recently, liquid biopsy, organoid-on-a-chip systems, patient-derived tumoroids (PDOs), CRISPR/Cas9 system, and the combination of AI and machine learning models have emerged as transformative models (Figure 3a–c). New standard-of-care-establishing trials, preliminary clinical trials, promising therapeutic approaches, functional precision, immune engineering, and immune repurposing are emerging as translational complements to molecular stratification (Table 1). Despite the challenges related to reproducibility, variability, cost, and ethics, combining these models with AI and machine-learning approaches may improve response prediction and increase the speed of new drug development [28], while advances in this field have led to the transition toward integrated precision oncology, in which histologic, clinical, and multi-omics data converge to inform individualized therapeutic decisions and define a new era of precision medicine for breast cancer [29].

Furthermore, based on the molecular information generated through multi-omics analyses, several platforms for dynamic analyses are now available to investigate important patients’ outcomes, such as liquid biopsy, which is a noninvasive tool for the analysis of ctDNA and/or circulating tumor cells (CTCs), providing real-time monitoring of molecular evolution, early detection of resistance, and identification of subclinical recurrence before obtaining radiologic evidence [60,61,62,63]. Clinical trials, such as c-TRAK TN, PADA-1, and EMERALD, have demonstrated the clinical utility of ctDNA for posttreatment surveillance and early selection of targeted therapies [62]. In addition, organoid-on-a-chip systems and PDOs have become known as high fidelity functional platforms that reproduce tumor architecture and aspects of the microenvironment, allowing for personalized testing of sensitivity and resistance to therapeutic agents. This procedure treatment strategy in locally advanced/metastatic unresectable disease on multiple treatment lines has had encouraging results, while highlighting the need for prospective validation [64].

Moreover, in 2012, Jinek et al. described how the bacterial CRISPR/Cas9 system could be repurposed as a genome-editing tool [65]. In breast cancer research, CRISPR/Cas9 has been widely used for disease modeling, discovery of target genes involved in tumor progression, assessment of drug sensitivity and immunotherapy response, evaluation of tumor fitness, and early diagnosis [66].

Phase I studies with immune engineering and immune repurposing approaches demonstrate effectiveness and biological signaling, but still with immature data for clinical approval. The first infusion of CRISPR-edited autologous T cells in humans established the feasibility of the manufacturing and administration with initial safety characterization in a small proof-of-concept cohort [67].

Altered cellular molecular components function as part of an integrated network within the complex tumor microenvironment, rather than as independent players, thereby driving cancer development and progression through dynamic interactions with the immune landscape of cancer.

Advances in tumor immunobiology have revealed that both the intrinsic characteristics of malignant cells and the composition and functional state of the tumor microenvironment critically influence disease progression and treatment response. This evolving knowledge has driven the development of a diverse array of immunotherapeutic and immune-based strategies, ranging from biomarkers that reflect antitumor immune activity to highly sophisticated targeted and cellular therapies. Collectively, these approaches aim to enhance immune recognition, to overcome tumor-induced immune evasion, and to deliver more precise and durable clinical benefits. In this context, the following sections summarize the key immune-related concepts and therapeutic modalities that are currently shaping cancer research and clinical practice.

## 2. Emerging Immunologic and Therapeutic Innovations in Breast Cancer

Contemporary breast cancer management is increasingly driven by interventions that either modulate the tumor microenvironment, exploit biomarker-defined vulnerabilities, or redirect immunity, with clinically meaningful gains most consistently demonstrated in randomized phase III clinical trials.

The TME constitutes a complex and heterogeneous ecosystem composed of multiple stromal components, including fibroblasts, mesenchymal cells, adipocytes, and diverse immune cell populations. This dynamic cellular milieu can foster the conditions that support tumor cell survival, growth, and proliferation [68,69]. Crosstalk among the TME constituents is largely mediated by the release of cytokines and growth factors, which play a pivotal role in modulating carcinogenesis, tumor progression, and therapeutic responsiveness in breast cancer [70]. Within this context, an in-depth understanding of the dynamic interplay between breast cancer cells and immune cells in the TME is essential, as it provides a critical foundation for the development of more precise and effective therapeutic strategies tailored to distinct breast cancer subtypes. 

To contextualize the translational relevance of these emerging therapeutic strategies, we will dissect the main clinical trials aligned with these new approaches, which explore immunological biomarkers, innovative immunity-based therapies, and combination strategies in different breast cancer settings, providing an overview of their design, primary outcome, and clinical results (Table 1). This synthesis aims to illustrate how advances in tumor immunology are being translated into clinical research and, ultimately, into practice-changing evidence.

### 2.1. Antiangiogenic Agents

Notably, early combinations of antiangiogenic agents with chemotherapy constituted the first clinical efforts to target the tumor microenvironment in breast cancer; however, these approaches mainly affected vascular support without actively reprogramming the tumor immune contexture.

Published in 2007, the phase III E2100 trial enrolled patients with the HER2-negative metastatic breast cancer disease and represented a clinical landmark by evaluating the benefit of adding bevacizumab, an anti-angiogenic monoclonal antibody targeting the tumor microenvironment, to standard treatment. The addition of bevacizumab to paclitaxel significantly prolonged progression-free survival (PFS) (11.8 vs. 5.9 months; HR 0.60; *p* < 0.001), highlighting that pathways not intrinsic to tumor cells can be therapeutically explored [30]. Based on these results, this therapeutic regimen was approved by the European Medicines Agency (EMA) and the Brazilian National Health Surveillance Agency (ANVISA) [71,72] for clinical use in patients with the same disease profile.

### 2.2. Tumor-Infiltrating Lymphocytes (TILs)

**Figure 4:**
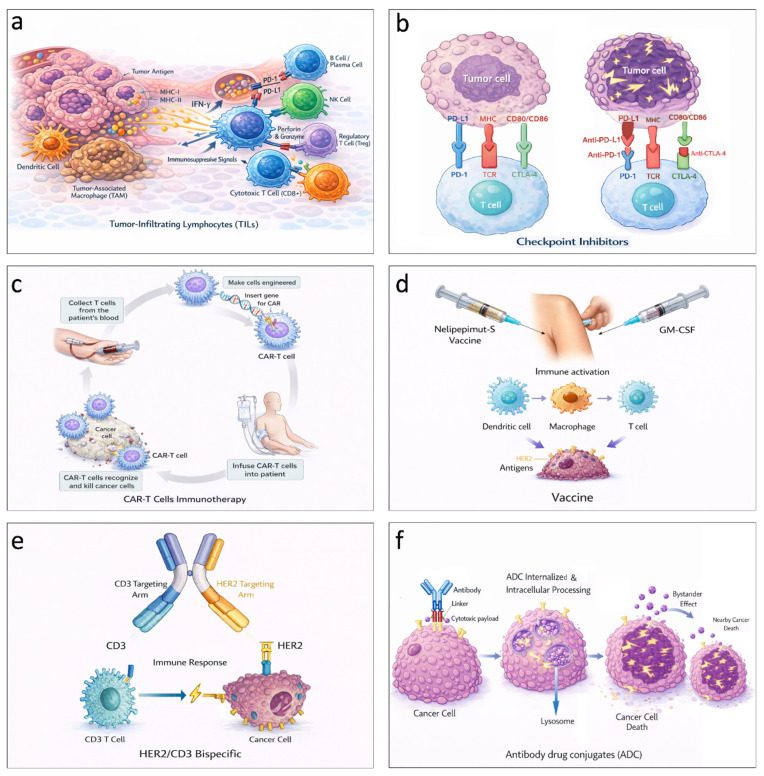
Immunotherapeutic and targeted delivery strategies in breast cancer. (**a**) Tumor-infiltrating lymphocytes (TILs). Stromal/intratumoral lymphocyte infiltration (e.g., CD8^+^ cytotoxic T cells, CD4^+^ T cells, NK cells, B cells) reflecting pre-existing antitumor immunity; in TNBC, higher TIL levels are associated with improved prognosis and increased sensitivity to systemic therapy. (**b**) Immune checkpoint inhibition. Blockade of PD-1/PD-L1 (and, conceptually, CTLA-4) to restore T-cell effector functions, to enhance tumor cell killing, and to overcome adaptive immune resistance driven by checkpoint signaling within the tumor microenvironment. (**c**) CAR T-cell therapy. Autologous T cells engineered to express a chimeric antigen receptor (CAR) enabling antigen-directed recognition and cytotoxic elimination of tumor cells independent of MHC, with ongoing optimization to address solid-tumor barriers (trafficking, antigen heterogeneity, immunosuppression). (**d**) Therapeutic cancer vaccines. Antigen-specific priming (e.g., peptide vaccines such as nelipepimut-S with GM-CSF as an immune adjuvant) to expand tumor-reactive T-cell responses and to support immune memory, often explored in combination strategies. (**e**) Bispecific antibodies (e.g., HER2/CD3). Dual binding to a tumor antigen and CD3 on T cells to physically bridge immune cells to tumor cells, triggering T-cell activation and redirected cytotoxicity at the tumor site. (**f**) Antibody–drug conjugates (ADCs). Antibody-mediated delivery of a cytotoxic payload via a linker, illustrating antigen binding, internalization, intracellular payload release, and potential bystander killing of neighboring antigen-low/negative cells depending on payload permeability and linker chemistry. Abbreviations: TILs, tumor-infiltrating lymphocytes; TNBC, triple-negative breast cancer; NK, natural killer; PD-1, programmed cell death protein 1; PD-L1, programmed death-ligand 1; CTLA-4, cytotoxic T-lymphocyte associated protein 4; CAR, chimeric antigen receptor; MHC, major histocompatibility complex; GM-CSF, granulocyte–macrophage colony-stimulating factor; ADC, antibody–drug conjugate. Created in OpenAI (2026) ChatGPT/DALL-E3 (version 5.2) on 10 February 2026, https://openai.com/.

TILs are pivotal immune components representing a heterogeneous population of mononuclear immune cells within the tumor microenvironment, functioning as primary indicators of host-mediated immunosurveillance and anticancer activity (Figure 4a). Therefore, recognition of the role of TILs in tumor immunogenicity and in the interactions between the immune system and the tumor microenvironment as prognostic and predictive biomarkers has supported the development of targeted immunotherapies [31,73,74]. The association of TILs through a multivariate statistical model in a pooled analysis of 3771 breast cancer patients treated with neoadjuvant therapy showed a prediction response to neoadjuvant chemotherapy with an increased TIL concentration, in luminal–HER2-negative, HER2-positive, TNBC subtype, and with a survival benefit in HER2-positive [31]. On the other hand, TILs increased concentration was an adverse prognostic factor in luminal-HER2-negative evidencing a different biology of the immunological infiltrate in this subtype [31].

In HER2+ breast cancer, TILs correlate with a pathological complete response (pCR) and survival post-trastuzumab/pertuzumab, with trends in hormone receptor-positive subsets of NeoSphere, TRYPHAENA trials [75,76,77,78,79]. In the TRAIN-2 trial, a sub-study identified TILs ≥ 60% as a rare (~12%) but powerful prognostic marker in stage II–III HER2+ breast cancer. These patients achieved 100% 3-year invasive disease-free survival (iDFS), independent of pCR or anthracycline use [80].

In TNBC, high stromal TILs (average 15–25%) were strongly prognostic: every 10% increase was associated with improved iDFS (HR 0.87), distant disease-free survival (DDFS) (HR 0.83), and overall survival (OS) (HR 0.84) in early-stage disease post-adjuvant anthracycline/taxane; TILs ≥ 30% in node-negative cases yielded 3-year OS 99% vs. 95% [81]. Lymphocyte-predominant breast cancer (LPBC, stromal TILs ≥ 50–60%) showed superior outcomes despite high grade [82]. The predictive value was evident in neoadjuvant settings (higher pCR rates), and metastatic TNBC (enhanced immunotherapy response) [83] and luminal breast cancer showed low TILs and minimal prognostic impact, while single hormone receptor-positive tumors displayed distinct patterns requiring further delineation. In ductal carcinoma in situ (DCIS), TILs had ambiguous prognostic significance. No specific toxicities were linked to the TIL assessment (morphological biomarker) [84].

TILs can specifically recognize tumor antigens, including neoantigens [85], providing a rationale for adoptive TIL therapy, as supported by a proof-of-concept report by Zacharakis et al. in 2018, where they showed a patient with chemorefractory HR-positive metastatic breast cancer achieved complete, 22-month durable regression after receiving mutation-specific TILs combined with IL-2 and checkpoint blockade, offering a promising new immunotherapy [86].

Given the intrinsic aggressiveness of TNBC, adjuvant chemotherapy is frequently recommended even for small stage IA–IB tumors, although international consensus guidelines show heterogeneity in recommendations for T1a and T1b disease. The St. Gallen International Expert Consensus generally advises adjuvant chemotherapy for T1b and larger TNBC [87], while many guidelines, including ESMO, recommend against routine chemotherapy for very early tumors (e.g., T1aN0), highlighting the need for individualized risk stratification [88]. Recognizing this uncertainty, biomarkers that can reliably guide treatment decisions and identify patients who may safely avoid overtreatment are of paramount importance.

Tumor-infiltrating lymphocyte levels have been demonstrated to reflect basal tumor immunogenicity and to carry an independent prognostic value in early TNBC, with higher TIL levels associated with improved outcomes, thus having great potential for de-escalation strategies in future clinical trials [81].

### 2.3. Checkpoint Inhibition

Adaptive immune homeostasis is regulated by immune checkpoint molecules, notably programmed death-ligand 1 (PD-L1), programmed death-1 (PD-1) and cytotoxic T-lymphocyte associated protein 4 (CTLA-4) (Figure 4b). The binding of PD-1 to PD-L1 on T lymphocytes can suppress effector T-cell activity and enhance regulatory T-cell (Treg) activity, mediating immunosuppression. Tumor cells may exploit this pathway to evade immune surveillance; elucidation of this mechanism has enabled the development of anti-PD-1 and anti-PD-L1 checkpoint inhibitors with clinically relevant effects on breast cancer, particularly in the TNBC disease [89].

In parallel, CTLA-4 acts at an earlier stage of T-cell activation by competitively inhibiting CD28-mediated costimulatory signaling through its interaction with CD80 and CD86 on antigen-presenting cells, leading to the attenuation of T-cell priming and expansion. Blockade of CTLA-4 enhances T-cell activation and proliferation, complementing PD-1/PD-L1 inhibition and providing a mechanistic rationale for combined checkpoint blockade strategies [90].

Early clinical trials evaluating CTLA-4 blockade in breast cancer explored both hormone receptor-positive (HR+/HER2−) and TNBC tumor settings, primarily in phase I or phase I/II designs, with a focus on safety, immune activation, and preliminary efficacy.

The combination of tremelimumab plus exemestane was investigated in a phase I trial involving patients with metastatic HR+/HER2− breast cancer. This study demonstrated that CTLA-4 inhibition could be safely combined with endocrine therapy, with manageable immune-related adverse events consistent, and with the known toxicity profile of CTLA-4 blockade. However, objective responses were rare, and the clinical benefit was limited, suggesting that immune checkpoint inhibition alone is insufficient to overcome the relatively low immunogenicity and immunosuppressive tumor microenvironment characteristic of luminal breast cancer. These findings underscored the biological constraints of applying CTLA-4 blockade in the HR+ disease without more potent immune-priming strategies [32].

A different biological rationale was explored in a phase I neoadjuvant “window-of-opportunity” study combining ipilimumab with tumor cryoablation in early-stage breast cancer. In this setting, cryoablation was used as an in situ vaccine to induce antigen release, followed by CTLA-4 inhibition to amplify T-cell priming. The study demonstrated increased intratumoral immune infiltration, expansion of activated T-cell populations, and systemic immune responses, supporting proof of immune modulation. Nevertheless, the trial was not designed to assess oncologic efficacy, and no conclusions regarding recurrence reduction or survival could be drawn. While biologically compelling, this approach remains experimental and highlights the gap between immune activation and clinically meaningful outcomes [33].

In more immunogenic subtypes, combined immune checkpoint blockade has shown more promising signals. A phase I/II trial of ipilimumab plus nivolumab in advanced or metastatic metaplastic TNBC demonstrated evidence of antitumor activity, including objective responses in a subset of heavily pretreated patients. The dual CTLA-4/PD-1 inhibition resulted in enhanced immune activation compared with monotherapy, but at the cost of increased immune-related toxicity. Although response rates were modest, these findings suggested that TNBC may be more susceptible to intensified immune checkpoint strategies, consistent with its higher baseline levels of tumor-infiltrating lymphocytes and genomic instability [34].

Similarly, a basket-type phase II clinical trial, published in 2024, evaluated the same therapeutic combination of ipilimumab plus nivolumab in advanced solid tumors, including breast cancer, confirming the feasibility and biological activity of dual checkpoint inhibition mainly in patients with high tumor mutational load (≥10 mutations per megabase). The adverse events were not different from those already reported in the literature, but toxicity remained a major limiting factor, reinforcing the need for careful patient selection and biomarker-driven approach [35].

Collectively, these early-phase studies have demonstrated that CTLA-4-based strategies are biologically active in breast cancer but clinically constrained. In the HR+/HER2-negative disease, limited immunogenicity translates into marginal efficacy despite immune engagement. In TNBC, although dual checkpoint inhibition can induce responses, toxicity and modest efficacy preclude broad clinical application outside clinical trials.

In contrast to exploratory, biologically driven early-phase studies evaluating CTLA-4 blockade, either alone or in combination with other immune checkpoint inhibitors, trials assessing the combination of chemotherapy with immune checkpoint inhibitors, particularly atezolizumab and pembrolizumab, have been supported by robust, practice-defining clinical evidence in TNBC. This evidence is derived from the demonstrated benefits observed in randomized clinical trials, including KEYNOTE-522, KEYNOTE-355, and IMpassion130 [36,37,38,91].

The KEYNOTE-522 trial provided compelling evidence that the addition of pembrolizumab to neoadjuvant chemotherapy followed by adjuvant pembrolizumab significantly improves long-term survival in patients with high-risk early-stage TNBC. After a median follow-up of approximately 75 months, the 5-year event-free survival (EFS) was 81.2–81.3% with pembrolizumab plus chemotherapy compared to 72.2–72.3% with chemotherapy alone, corresponding to a hazard ratio (HR) for EFS of approximately 0.63–0.65; the 5-year OS rate was 86.6% versus 81.7% (HR for death 0.66; *p* = 0.0015), demonstrating a durable, clinically meaningful survival benefit [36,91].

In a metastatic setting, the KEYNOTE-355 trial showed that pembrolizumab plus chemotherapy significantly improved PFS in patients with PD-L1-positive TNBC (CPS ≥ 10), with a median PFS of 9.7 months versus 5.6 months for chemotherapy alone (HR 0.65), and also demonstrated improved OS in this subgroup compared with a placebo plus chemotherapy [37].

The IMpassion130 trial established atezolizumab plus nab-paclitaxel as a first-line option for PD-L1-positive metastatic TNBC by showing significant PFS benefits in the intention-to-treat population (median 7.2 vs. 5.5 months; HR 0.80) and a more pronounced benefit in the PD-L1-positive subgroup (median PFS 7.5 vs. 5.0 months; HR 0.62). While the OS improvement in the overall population did not reach statistical significance, exploratory analyses showed a clinically meaningful median OS advantage in PD-L1-positive patients (25.4 vs. 17.9 months; HR 0.67) [38,39,72].

Taken together, these results support the incorporation of immune checkpoint inhibitors with chemotherapy as the standard of care across the spectrum of TNBC, highlighting the critical role of PD-L1 biomarker selection and establishing pembrolizumab in early stages regardless of PD-L1 positivity and pembrolizumab or atezolizumab in metastatic PD-L1-positive settings.

Additional checkpoint inhibitor programs have extended the evidence base to anti-PD-L1 in neoadjuvant and metastatic trials. The GeparNuevo study is a randomized phase II placebo-controlled trial investigating the neoadjuvant addition of durvalumab to an anthracycline/taxane-containing chemotherapy in TNBC, which demonstrated a modest/non-significant pathological complete response rate (an absolute 9% gain from 44% to 53%), after a median follow-up of 43.7 months (range 4.9–56.1 months); a significantly longer 3-year invasive disease free survival (iDFS) was observed with the addition of durvalumab; 3-year iDFS 85.6% (95% CI 76.0% to 91.6%) with durvalumab and 77.2% (95% CI 66.3% to 85.0%) with placebo (HR 0.48 (95% CI 0.24–0.97), *p* = 0.036). [40], while the anit-PD-L1 toripalimab, in the TORCHLIGHT phase III trial improved outcomes in PD-L1-positive metastatic/recurrent TNBC (median PFS 8.4 versus 5.6 months; HR = 0.65, 95% CI 0.470–0.906, *p* = 0.0102), and the median OS was 32.8 versus 19.5 months (HR = 0.62, 95% CI 0.414–0.914, *p* = 0.0148) [41].

Building on the consistent clinical outcomes demonstrated in the KEYNOTE-522, KEYNOTE-355 and IMpassion130 trials, the GeparNuevo and TORCHLIGHT studies demonstrated that the benefit of immune checkpoint inhibitors in the neoadjuvant setting is largely independent of PD-L1 expression, while in the metastatic disease it is clearly dependent on PD-L1 status.

### 2.4. Chimeric Antigen Receptor T-Cell (CAR T-Cell) Therapy

Genetically engineered T lymphocytes expressing chimeric antigen receptors (CAR T-cells) (Figure 4c) have transformed therapy for hematologic malignancies and represent a precise and promising approach for solid tumors, including breast cancer [92]. However, key barriers include tumor heterogeneity, an immunosuppressive microenvironment, antigen expression in normal tissues (on-target/off-tumor toxicity), and limited CAR T-cell infiltration and persistence within tumors, as well as high costs [92].

CAR T-cell platforms have evolved through multiple structural and functional generations. In breast cancer, the leading targets under investigation include HER2, MUC-1, mesothelin, ROR1, and c-Met [93,94]. Clinical data remain sparse and immature, with no phase II/III completed trials in BC/TNBC [95].

Phase I studies have evaluated the efficacy and safety of intratumoral delivery of c-Met-redirected CAR T cells, demonstrating strong anticancer activity against TNBC with no toxicity greater than grade 1 [96]. Jaeger-Ruckstuhl et al. (2025) [97] conducted a first-in-human trial targeting receptor tyrosine kinase-like orphan receptor 1 (ROR1) using CAR T cells in patients with B-cell malignancies or advanced TNBC and non-small cell lung cancer (NSCLC). Treatment was generally well tolerated; cytokine release syndrome (CRS) occurred in 76% of patients (grade ≥ 2 in 48%), while immune effector cell-associated neurotoxicity syndrome (ICANS) was rare (9.5%), with all events limited to grade 1. In patients with solid tumors (TNBC, *n* = 10; NSCLC, *n* = 8), CAR T-cell expansion was highly variable and stratified as high (>50%), medium (3–50%), or low (<3%) for circulating CD8^+^ T cells. Tumor infiltration remained limited, and only 1 of 18 patients (5.5%) achieved a partial response. ROR1 immunohistochemistry staining (>20% of tumor cells) was used as an inclusion biomarker; however, subcellular localization patterns did not clearly predict the clinical response [97]. Similarly, a pilot trial investigating CAR-RNA c-Met T cells in heavily pretreated patients, including those with metastatic TNBC, reported no grade ≥ 3 toxicities but limited clinical efficacy. Among the seven treated patients, the best response was a stable disease in four patients, while three patients experienced a progressive disease, highlighting the persistent barriers to CAR T-cell therapy in solid tumors [42]. Collectively, these trials have demonstrated the feasibility and safety of CAR T-cell therapy in breast cancer, albeit with limited antitumor activity. Notably, no high-grade CRS or neurotoxicity was reported in breast cancer trials, in contrast to observations in hematologic malignancies. Furthermore, the expression of tumor-associated antigens (e.g., HER2, EGFR, MSLN) appears to influence tumor targeting, while features of the tumor microenvironment, including PD-L1 expression, tumor-infiltrating lymphocytes, and myeloid-derived suppressor cells, affect the therapeutic response [95]. Effective management of treatment-related toxicities is critical for clinical success; in this regard, adverse events, such as grade 1–2 CRS and ICANS, can be managed with tocilizumab and corticosteroids [98]. Nevertheless, proposed predictive biomarkers remain insufficiently validated.

Taken together, although CAR T-cell therapy has shown promising preclinical and early clinical activity in breast cancer, its clinical application remains limited due to the current lack of robust supporting evidence. Consequently, the translation of CAR T-cell therapy into routine clinical practice will require additional well-designed clinical trials and deeper mechanistic investigations aimed at improving safety and overcoming the biological and technical challenges inherent to this therapeutic strategy [99].

### 2.5. Vaccines

Breast cancer immunotherapy can be understood within the cancer immunoediting paradigm, which delineates the elimination, equilibrium, and escape phases. During the elimination phase, innate immune effectors (macrophages, NK cells, and dendritic cells) prime adaptive immune responses through CD8^+^ cytotoxic T lymphocytes and CD4^+^ helper T cells that recognize tumor antigens presented by MHC class I and class II molecules. Tumor-specific antigens (TSAs), including neoantigens derived from somatic mutations and oncoviral antigens, elicit robust immune responses due to the absence of central tolerance, whereas tumor-associated antigens (TAAs), such as overexpressed HER2, MUC1, CEA, and NY-ESO-1, induce weaker, tolerance-prone immunity. Tumor escape mechanisms include the downregulation of MHC molecules, the upregulation of immune checkpoint proteins (PD-L1, CTLA-4), the recruitment of immunosuppressive cells (regulatory T cells, myeloid-derived suppressor cells, and tumor-associated macrophages), and shifts in the cytokine milieu (TGF-β, IL-10) that promote T-cell anergy and exhaustion. Breast cancer vaccines aim to counteract immune escape by enhancing antigen-specific CD8^+^ and CD4^+^ T-cell responses, improving dendritic cell cross-presentation, and generating long-lasting memory T cells [100]. Therapeutic cancer vaccines, first developed in the 1990s, represent a promising immunotherapy strategy capable of targeting tumor-associated antigens. The principal targets of peptide-based vaccines include MUC-1, EphA receptors, survivin, SART3, and nelipepimut-S, an immunogenic peptide derived from HER2 [101]. 

Peptide vaccines targeting MHC class I epitopes represent a common immunotherapeutic strategy in breast cancer. Following injection, peptides are taken up and processed by antigen-presenting cells (APCs), leading to the priming of effector T cells that recognize tumor-associated antigens such as HER2 [102]. Although short peptides are cost-effective, stable, and easy to manufacture, their clinical applicability is limited by HLA restriction, which confines eligibility to specific patient subsets [103,104]. In addition, short peptides often fail to adequately activate CD4^+^ helper T cells, resulting in weak and transient immune responses [105]. To overcome these limitations, synthetic long peptides (23–45 amino acids) have been developed. These peptides encompass multiple MHC class I and class II epitopes, enabling more effective T-cell activation and generating stronger and more durable antitumor immunity [106].

Among peptide-based vaccines, nelipepimut-S (NP-S) has the most extensive clinical evidence. Its combination with granulocyte–macrophage colony-stimulating factor (GM-CSF) (Figure 4d) progressed from phase I–II studies in patients with high-risk, early-stage breast cancer [107] to phase III PRESENT adjuvant trial. In this study, patients received either NP-S (1000 μg) plus GM-CSF (250 μg) or a placebo plus GM-CSF monthly for six months, followed by administration every six months up to 36 months. An interim analysis at a median follow-up of 16.8 months revealed no significant difference in disease-free survival (DFS) between the two arms. Notably, in the NP-S group, imaging detected 54.1% of recurrence events in asymptomatic patients, compared with 29.2% in the placebo group (*p* = 0.069); the trial was subsequently terminated early [43].

Dendritic cell (DC)-based vaccines have also been investigated, given the central role of DCs as professional antigen-presenting cells. DCs are critical for reversing immunosuppression in both HER2-positive and TNBC. Ex vivo matured DCs loaded with tumor antigens or neoantigens promote CD8^+^ T-cell priming and CD4^+^ Th1 polarization through interleukin-12p70 secretion. While breast tumors evade immune surveillance via PD-L1 upregulation and recruitment of inhibitory immune populations such as regulatory T cells and myeloid-derived suppressor cells, DC vaccines counteract these mechanisms by inducing interferon-γ-driven responses and synergizing with chemotherapy-induced immunogenic cell death [108].

Clinical studies of DC vaccines in breast cancer have demonstrated favorable safety profiles and preliminary efficacy across multiple subtypes. Phase I/II trials combining autologous DC vaccination with neoadjuvant chemotherapy (NAC) restored T-cell reactivity and were well tolerated [109]. In HER2-negative breast cancer, the addition of DC vaccines to NAC increased pCR rates, particularly in PD-L1-negative tumors, and significantly reduced tumor burden [110]. In the HER2-positive disease, preclinical models and phase I trials have demonstrated synergistic activity between DC vaccination and anti-PD-1 therapy, underscoring the importance of CD4^+^ T cells in mediating survival benefits [44,111,112]. Moreover, anti-HER2 DC vaccines elicited strong tumor-specific T-cell responses, with higher pCR rates observed in DCIS compared with invasive carcinoma [113]; this effect was further enhanced by concomitant anti-estrogen therapy in estrogen receptor-positive tumors [114]. In TNBC, DC vaccines combined with NAC increased intratumoral CD8^+^ T-cell infiltration, suggesting potential clinical benefit [115]. Despite these encouraging findings, DC-based vaccines have not yet advanced to a phase III clinical evaluation [116]. Overall, while DC vaccines show considerable promise, their antitumor efficacy appears highly dependent on synergistic combinations with chemotherapy, immune checkpoint inhibitors, or adoptive immunotherapies to optimize clinical outcomes.

Carbohydrate antigens expressed on breast cancer cells have also been explored as vaccine targets. Sialyl-Tn (STn), a disaccharide carbohydrate associated with MUC-1, is uniquely expressed on the surface of multiple carcinomas, including breast cancer, making it an attractive vaccine candidate [117]. In a phase III clinical trial, patients receiving endocrine therapy combined with an STn-keyhole limpet hemocyanin (KLH) conjugate vaccine (Theratope) demonstrated improved overall survival (37 months) compared with those receiving KLH alone (31 months). Despite this six-month survival benefit, the clinical applicability of the study is limited by the absence of molecular subtype stratification. In particular, the number of patients with TNBC was not reported, precluding the assessment of vaccine efficacy in this subgroup [118]. 

Messenger RNA (mRNA) vaccine technology, accelerated by platforms developed during the COVID-19 pandemic, represents a promising active immunotherapy for breast cancer, particularly for immunologically “cold” tumors. mRNA vaccines enable the rapid and scalable production of personalized neoantigen- or shared tumor-associated antigen-encoding constructs, typically delivered via optimized lipid nanoparticle (LNP) formulations. These platforms elicit potent and durable CD8^+^ and CD4^+^ T-cell responses and may overcome immune tolerance and tumor microenvironment-mediated suppression more effectively than conventional vaccine approaches [119]. A multi-omics preclinical study integrating TCGA, METABRIC, and single-cell sequencing data identified three tumor-associated antigens (TAAs), CD74, IRF1, and PSME2, that were associated with improved patient prognosis and increased infiltration of antigen-presenting cells, including B cells and dendritic cells, within the tumor microenvironment [120]. Early-phase clinical data have further suggested a synergistic potential between mRNA vaccines and immune checkpoint blockade or anti-HER2 therapies, enhancing TIL recruitment and clinical activity. Despite ongoing challenges related to immunogenicity and tumor microenvironment barriers, the flexibility, favorable safety profile, and multi-epitope targeting capacity of mRNA vaccines position them as promising candidates for biomarker-driven combination trials in residual or metastatic disease, particularly in low-mutational burden solid tumors [121]. 

Overall, breast cancer vaccine research remains an active field, encompassing multi-epitope formulations, neoantigen-based DNA platforms, and mRNA vaccines, frequently in combination with other immunotherapeutic modalities to enhance immunogenicity. Although clinical efficacy has been variable and no vaccine has yet received regulatory approval for breast cancer treatment, the evolving evidence underscores the substantial therapeutic potential of cancer vaccines and supports their continued investigation in future clinical studies [116,117,118,119,120,121,122,123,124,125]. 

### 2.6. Bispecific Antibodies

Bispecific antibodies (BsAbs) represent one of the most recent and innovative immunotherapeutic approaches for the treatment of breast cancer. These agents combine two distinct mechanisms of action within a single molecule, either by redirecting T cells toward tumor cells (e.g., HER2/CD3) or by simultaneously targeting two immune or oncogenic pathways to limit tumor escape mechanisms [126]. BsAbs are generated through advanced molecular engineering using multiple platforms, including Fc-engineered constructs, DuoBody^®^, BiTE^®^, and CrossMab technologies [127]. By concurrently engaging TAAs, such as HER2, HER3, EGFR, and TROP2, on cancer cells and immune effector receptors (e.g., CD3 on T cells or CD16 on natural killer cells and macrophages) (Figure 4e), BsAbs redirect cytotoxic activity or inhibit complementary signaling pathways, such as HER2/HER3 dimerization [127]. Most BsAbs remain in the early stages of clinical development and are frequently evaluated in combination with cancer vaccines, adoptive cell therapies, or immune checkpoint inhibitors to enhance therapeutic efficacy [128]. 

Clinical investigations of BsAbs in breast cancer have primarily focused on enhancing immune-mediated cytotoxicity through dual-antigen targeting. MDX-H210, a bispecific antibody that links FcγRI (CD64) on myeloid cells to HER2-expressing tumor cells, demonstrated an acceptable safety profile and biological activity, including cytokine release (IL-6 and TNF-α) and neutrophil-mediated cytotoxicity, when administered in combination with granulocyte colony-stimulating factor (G-CSF). However, objective clinical responses were limited in heavily pretreated patients with metastatic disease [129,130,131,132].

By contrast, KN026, a bispecific antibody targeting two distinct HER2 epitopes, exhibited a manageable safety profile and encouraging clinical activity, with an objective response rate (ORR) of 28.1%. Importantly, co-amplification of CDK12 emerged as a promising predictive biomarker of response to KN026, with patients harboring CDK12 co-amplification demonstrating superior outcomes compared with those without co-amplification (ORR: 50% vs. 0%; median PFS: 8.2 vs. 2.7 months; *p* = 0.05 and 0.04, respectively) [45]. Subsequent analyses have confirmed that co-amplification of HER2 and CDK12 significantly enhanced therapeutic response, effectively doubling the response rate relative to non-amplified tumors [133]. Additionally, immune consolidation strategies using HER2-targeted “activated T cells” (BATs) armed with anti-CD3 × anti-HER2 BsAbs have demonstrated clinical potential. Trials have reported that improved OS bispecific T-cell repurposing approaches also demonstrate early clinical activity: HER2 bi-activated T cells showed no dose-limiting toxicities (DLTs), and disease stabilization in 59.1% of patients at 14.5 weeks [134] in both HER2-positive and TNBC patients correlated with increased Th1 cytokine production and antigen-specific T-cell responses [134,135]. Similarly, ertumaxomab demonstrated objective responses and disease control in a small phase I study [46]. Collectively, these findings highlight the capacity of bispecific antibodies to reprogram the tumor immune microenvironment and to enhance antitumor immunity, particularly when guided by predictive biomarkers or integrated with adoptive cell therapies.

### 2.7. Antibody–Drug Conjugates (ADCs)

ADCs are targeted antineoplastic agents composed of an antibody, a linker, and a cytotoxic payload [136]. The antibody provides target specificity, while the linker helps ensure that the payload is released after the conjugate reaches the target cell (Figure 4f) [137].

The antitumor activity of ADCs is carried out through three mechanisms: (1) targeted cytotoxicity in antigen-expressing tumor cells, (2) bystander killing of neighboring malignant cells, and (3) immunomodulatory remodeling of the tumor microenvironment [138]. These immunoregulatory effects are largely attributed to payload-induced immunogenic cell death, enhanced antigen presentation [139,140], and synergy with immunotherapy, supporting the development of immuno-ADCs for the treatment of breast cancer [141,142]. Ongoing optimization includes the development of bispecific ADCs and dual-payload ADCs [143].

Antibody–drug conjugates have redefined the treatment of breast cancer by enabling the targeted delivery of highly potent cytotoxic agents to HER2-expressing tumor cells. In the HER2-positive disease, trastuzumab emtansine (T-DM1) was the first ADC to demonstrate a clear clinical benefit. The phase III EMILIA trial showed significant improvements in the PFS (9.6 months with T-DM1 versus 6.4 months with lapatinib plus capecitabine (HR 0.65; 95% CI, 0.55 to 0.77; *p* < 0.001) and the OS median with T-DM1 compared with lapatinib plus capecitabine (30.9 months vs. 25.1 months; HR 0.68; 95% CI, 0.55 to 0.85; *p* < 0.001) [47].

In the early-stage disease, the KATHERINE trial assessed T-DM1 compared with trastuzumab as an adjuvant treatment in patients with the HER2-positive with residual invasive disease in the breast or axilla after neoadjuvant systemic treatment with taxane-based chemotherapy and trastuzumab, with a median follow-up of 8.4 years, trastuzumab emtansine sustained the improvement in iDFS over trastuzumab (unstratified HR for invasive disease or death 0.54. The seven-year OS was 89.1% with trastuzumab emtansine and 84.4% with trastuzumab [144].

The next-generation ADC trastuzumab deruxtecan (T-DXd) further shifted treatment paradigms. In metastatic HER2-positive breast cancer, the phase III DESTINY-Breast03 trial demonstrated the superiority of T-DXd over T-DM1, with a marked improvement in the PFS (28.8 months 95% CI 22.4–37.9) with trastuzumab deruxtecan and 6.8 months with trastuzumab emtansine HR 0.33; 95% CI 0.26–0.43]; *p* < 0.0001), a statistically significant and clinically meaningful OS improvement versus T-DM1, with a reduction in the risk for death of approximately 36% (HR 0.64; 95% CI 0.47–0.87; *p* = 0.0037) [48]. The survival benefit observed in this study, never before seen in other studies with similar profiles, and the acceptable toxicity support the use of T-DXd as a standard second-line treatment for patients with HER2-positive metastatic breast cancer.

Beyond HER2-positive tumors, T-DXd has expanded the therapeutic options for tumors with low levels of HER2 expression (HER2 IHC 1+ or IHC 2+ and ISH-negative). The phase III DESTINY-Breast04 trial established T-DXd as the standard therapy in HER2-low metastatic breast cancer, demonstrating significant improvements in the PFS (9.9 months in the trastuzumab deruxtecan group and 5.1 months in the physician’s choice group; HR 0.50, *p* < 0.001) and OS (23.4 months and 16.8 months; HR 0.64, *p* = 0.001) compared with chemotherapy [49].

This concept was extended by the DESTINY-Breast06 trial, a phase III trial involving patients with hormone receptor-positive metastatic breast cancer with low HER2 expression or ultralow HER2 expression (IHC 0 with membrane staining) who received prior endocrine therapy but not chemotherapy for metastatic disease. The trial met its primary endpoint by showing an improved PFS with T-DXd versus chemotherapy. For patients with the HER2-low disease, the median progression-free survival was 13.2 months (95% CI, 11.4 to 15.2) in the trastuzumab deruxtecan group and 8.1 months (95% CI, 7.0 to 9.0) in the chemotherapy group (HR 0.62; 95% CI, 0.52 to 0.75; *p* < 0.001); the results were consistent in the exploratory HER2-ultralow population. Data for overall survival were immature [50]. Despite the positive results, we highlight the difficulties in accurately identifying low and ultra-low levels of HER2 expression and differentiating them from samples that do not express HER2 (IHC 0, no staining observed), and the need for further assay optimization and training in HER2 IHC 0 and HER2-ultralow scoring, a role in which AI could significantly contribute.

In light of the robust efficacy of T-DXd demonstrated after first-line therapy in metastatic HER2-positive breast cancer, there is a strong rationale for evaluating its use earlier in the disease course. Accordingly, the DESTINY-Breast09 and DESTINY-Breast05 trials were designed to investigate T-DXd in first-line metastatic and adjuvant settings, respectively [51,52].

The phase III DESTINY-Breast09 trial enrolled patients to a first-line metastatic HER2-positive setting. They were randomly assigned in a 1:1:1 ratio to receive trastuzumab deruxtecan plus pertuzumab, trastuzumab deruxtecan plus placebo, or a taxane, plus trastuzumab and pertuzumab. The trial met its primary endpoint, showing that T-DXd plus pertuzumab significantly prolonged the PFS compared with trastuzumab, pertuzumab, and a taxane (median PFS 40.7 with trastuzumab deruxtecan plus pertuzumab vs. 26.9 months with a taxane plus trastuzumab and pertuzumab; HR 0.56; 95% CI, 0.44 to 0.71; *p* < 0.00001), supporting a potential paradigm shift toward ADC-based regimens in the upfront setting [51].

To evaluate the benefit of T-DXd in patients with the high-risk, early-stage HER2-positive disease, the phase III DESTINY-Breast05 study was designed, which randomly assigned patients with HER2-positive breast cancer with residual invasive disease and node-positive disease at surgery or inoperable disease at diagnosis to receive adjuvant T-DXd or T-DM1. T-DXd significantly improved invasive disease-free survival (iDFS) compared with T-DM1, with invasive disease events or death occurring in 6.2% of patients in the T-DXd group vs. 12.5% in the T-DM1 group (HR 0.47; 95% CI, 0.34–0.66; *p* < 0.001); the 3-year iDFS was 92.4% and 83.7%, respectively [52], reinforcing the role of ADCs even in curative-intent settings.

Complementing these efficacy-focused trials, the phase IIIb/IV DESTINY-Breast12 trial was undertaken to evaluate the safety and clinical and intracranial activity of T-DXd in a broader, real-world HER2-positive cohort, including patients with non-brain metastases and stable or active brain metastases, a historically underrepresented group in randomized trials. Primary endpoints were PFS in a brain metastases (BMs) cohort and ORR in a non-BMs cohort. In the BMs cohort, the 12-month PFS was 61.6% (95% CI; 54.9–67.6) and the 12-month central nervous system PFS (CNS PFS) was 58.9% (95% CI: 51.9–65.3). In the non-BMs cohort, the ORR was 62.7% (95% CI: 56.5–68.8) [53]. These initial results demonstrated clinically significant systemic and intracranial activity with a safety profile consistent with previous studies, supporting the efficacy of T-DXd in various clinical breast cancer settings.

T-DXd has demonstrated superior efficacy over earlier antibody–drug conjugates owing to its optimized molecular architecture, characterized by a high drug-to-antibody ratio, a cleavable linker, and a potent, membrane-permeable topoisomerase I inhibitor payload. This design enables efficient intracellular payload release and a pronounced bystander effect, thereby mitigating the impact of intratumoral HER2 heterogeneity. Nonetheless, despite these advances, many patients with advanced HER2-positive breast cancer ultimately develop resistance and experience disease progression, underscoring the unmet need for next-generation ADCs incorporating novel cytotoxic payloads, alternative molecular targets, and improved site-specific conjugation strategies.

Other next-generation HER2-targeted ADCs further bolster the class’s momentum, with PFS gains reported for the phase III TULIP trial which evaluated trastuzumab duocarmazine (SYD985/T-Duo) versus physician’s choice chemotherapy in patients with unresectable locally advanced or metastatic HER2-positive breast cancer who had progressed after at least two prior HER2-targeted therapies, including T-DM1. The study met its primary endpoint of PFS centrally reviewed; the median PFS was 7.0 months (95% CI, 5.4 to 7.2) with T-Duo versus 4.9 months with physician’s choice (HR 0.64; 95% CI, 0.49 to 0.84; *p* = 0.002) representing a 36% reduction in the risk of progression or death. The median OS favored T-Duo numerically 20.4 vs. 16.3 months (HR 0.83; 95% CI, 0.62 to 1.09; *p* = 0.153) but did not reach statistical significance at the first analysis [54]. Even though a clinical benefit was observed in this population, heavily pretreated treatment with T-Duo should be considered only after further safety studies, since there was a prevalence of ocular toxicity, leading to a higher discontinuation rate in the T-Duo group.

Continuing to explore the HER2 blockade after disease progression following prior HER2-targeted therapy, the Chinese phase III ACE-Breast-02 trial evaluated ARX788, a homogeneous, site-specific antibody–drug conjugate combining an anti-HER2 antibody with a potent tubulin inhibitor versus lapatinib plus capecitabine (LC) in patients with HER2-positive advanced breast cancer who had progressed after one line of trastuzumab. The primary endpoint, progression-free survival, was significantly prolonged with ARX788 (11.3 months; 95% CI, 8.4–13.8) compared with LC (8.2 months; 95% CI, 6.9–8.7), as assessed by a blinded independent central review (HR 0.64; *p* = 0.0006) [55].

These results demonstrate the superior PFS benefit of ARX788 over standard LC in the advanced HER2-positive disease, supporting its potential as an effective therapeutic option. Nevertheless, given its distinct ADC structure and payload, careful characterization of its safety profile and adverse-event spectrum, particularly in comparison with currently approved ADCs, remains essential.

Another important extension of the ADC technology is targeting the HER3 axis in patients with central nervous system (CNS) involvement, a setting of high unmet need due to historically poor outcomes and limited treatment options.

The basket phase II TUXEDO-3 trial evaluated patritumab deruxtecan (HER3-DXd) a HER3-directed ADC in patients with metastatic solid tumors including breast cancer with active brain metastases or leptomeningeal disease, an especially challenging clinical scenario. In the breast cancer cohort, the study met its primary endpoint of intracranial response, with 24% of patients having intracranial responses irrespective of the breast cancer subtype (ORR 23.8%, 95% CI 8.2–47.1) among evaluable patients with active brain metastases, demonstrating notable central nervous system activity across subtypes, including the HER2-positive and triple-negative disease. Notably, in the leptomeningeal cohort, the 3-month OS rate was approximately 69.6%, exceeding the predefined threshold for clinical significance, with a median OS of 10.5 months in this historically poor-prognosis population [56]. These findings underscore the potential of HER3-DXd to penetrate the blood–brain barrier and to elicit clinically meaningful responses in CNS/leptomeningeal disease, a promising addition to systemic treatment strategies in patients with metastatic breast cancer and CNS involvement.

Beyond HER2-directed ADCs, the clinical success of this therapeutic platform has also extended to HER2-independent targets, most notably TROP2, further broadening the role of ADCs in breast cancer. Sacituzumab govitecan, a TROP2-directed ADC delivering the topoisomerase I inhibitor SN-38, has also shown robust efficacy in triple-negative breast cancer (TNBC). The phase III ASCENT-03 trial evaluated sacituzumab govitecan as a first-line therapy in patients with previously untreated metastatic TNBC who were not candidates for PD-1/PD-L1 inhibitors. The study met its primary endpoint, demonstrating a statistically significant improvement in the PFS compared with the standard chemotherapy median PFS of 9.7 months (95% CI, 8.1 to 11.1) with sacituzumab govitecan and 6.9 months (95% CI, 5.6 to 8.2) with chemotherapy stratified HR for disease progression or death, 0.62 (95% CI, 0.50 to 0.77; *p* < 0.001), with consistent benefit across prespecified subgroups. The OS data were immature at the primary analysis cut-off [57], complementing the scenario of untreated TNBC. The phase III ASCENT-04/KEYNOTE-D19 trial assessed sacituzumab govitecan plus pembrolizumab versus standard chemotherapy plus pembrolizumab for PD-L1-positive metastatic TNBC. This study also met its primary endpoint, with the combination significantly prolonging the median PFS (11.2 vs. 7.8 months; HR 0.65; *p* < 0.001). The objective response rate and durability of response were both higher with sacituzumab govitecan plus pembrolizumab, and while the OS data remain immature, early trends favor improved outcomes with the ADC–immunotherapy combination [58].

In addition to assessing its benefit in metastatic TNBC, regardless of PD-L1 status, sacituzumab govitecan was also evaluated in a heavily pretreated population with HER2-negative, hormone receptor-positive (HR+) breast cancer. This group was selected due to its higher potential for proliferation independent of hormonal pathways, as demonstrated in the phase III TROPiCS-02 study [145].

The phase 3 TROPiCS-02 trial randomized patients with confirmed HR+ and HER2 negative locally recurrent inoperable or metastatic breast cancer who had received at least one previous endocrine therapy, a taxane, and a CDK4/6 inhibitor in any setting and two to four previous chemotherapy regimens for metastatic disease. The primary endpoint was a PFS which was significantly improved with sacituzumab govitecan compared with chemotherapy, with a median of 5.5 months vs. 4.0 months, respectively (HR 0.66; 95% CI; 0.53–0.83; *p* = 0.0003). The OS was also significantly longer in the sacituzumab govitecan group, with a median OS of 14.4 months (95% CI 13.0–15.7) compared with 11.2 months in the chemotherapy group (HR 0.79; 95% CI, 0.65–0.96; *p* = 0.020) [145]. These data support sacituzumab govitecan as a new treatment option for patients with pretreated, endocrine-resistant HR+ and HER2 negative metastatic breast cancer.

The phase III TROPION-Breast01 trial evaluated datopotamab deruxtecan (Dato-DXd) a TROP2-directed antibody–drug conjugate (ADC) vs. the investigator’s choice of standard chemotherapy in adults with inoperable or metastatic hormone receptor-positive (HR+) breast cancer that is HER2-low or HER2-negative, after prior endocrine therapy and at least one line of chemotherapy. The trial met its primary endpoint, with Dato-DXd significantly prolonging the PFS compared with chemotherapy (median PFS 6.9 vs. 4.9 months, HR 0.63; 95% CI, 0.52 to 0.76; *p* < 0.0001 by blinded independent central review), The confirmed ORR was higher with Dato-DXd (36.4%) than with chemotherapy (22.9%). In the final analysis, the OS did not reach statistical significance between the two arms (HR 0.84; 95% CI; 0.62 to 1.14), although use of subsequent ADCs was imbalanced and secondary efficacy measures (e.g., ORR, DCR, disease control) continued to favor Dato-DXd [59].

Collectively, these data have illustrated a translational trajectory in which the most mature, high-impact gains arise when biologic targeting (HER2, HER3, TROP2, PD-L1 status) is coupled to modalities capable of overcoming tumor heterogeneity and immune escape (ADCs, immune checkpoint inhibitors—ICIs), while emerging platforms (organoids, CRISPR, CAR-T, bispecifics) continue to define the next wave of individualized therapy through improved functional prediction, engineered immune potency, and rational combination strategies.

### 2.8. Digital Pathology, Advanced IHC, and Automated Analysis

From a pathological perspective, advances in breast cancer research depend on the quality of the material evaluated and on an accurate, timely diagnosis.

A digital pathological analysis in breast cancer begins with the systematic digitization of glass slides using whole-slide imaging (WSI) scanners, generating high-resolution files that can be viewed, annotated, and analyzed on workstations or online platforms. This transforms tissue features into numerical data and enables the AI-supported quantitative analysis of biomarkers [146], including estrogen receptor (ER), progesterone receptor (PR), HER2, and Ki-67, and histologic grade core elements of therapeutic decision-making but subject to interobserver variability.

In the context of Ki-67, recent studies involving dozens of pathologists have shown that AI assistance improves accuracy, increases interobserver agreement, and reduces reporting time by quantifying larger tissue areas or the whole slide, providing a more robust measure of proliferation [147].

More recently, HER2 IHC interpretation has shifted from a binary positive/negative framework to include HER2-low (IHC 1+ or 2+/IHC−) and ultralow categories, raising concerns about interobserver agreement and intratumoral heterogeneity [148,149].

Mitotic counting is an essential component of Nottingham grading and remains labor intensive, depends on observer experience, and is susceptible to errors due to fatigue or the selection of nonrepresentative areas; AI-based approaches can mitigate these limitations [150].

With WSI, AI algorithms can achieve the following:

- Automatically identify tumor nuclei (distinguishing them from the nuclei of inflammatory cells and fibroblasts, and artifacts);
- Classify each nucleus as positive/negative or according to an intensity scale (0, 1+, 2+, 3+);
- Generate expression heatmaps and global indices (e.g., Ki-67 positivity across the slide rather than only 500–1000 manually selected cells).

Traditionally, TILs are visually estimated by hematoxylin and eosin (H&E) staining and quantified in terms of the percentage of infiltrated tumor stroma, which is a subjective process. Digital pathology enables computational TIL assessment, in which the following steps occur:

- Algorithms segment the tumor and stroma and identify lymphocytes within the stroma on the basis of morphological features;
- Quantitative metrics (e.g., density, percentage of infiltrated area, and spatial distribution) are computed and correlated with clinical outcomes and therapeutic response.

Comparative studies have demonstrated good correlation between computational and manual TIL scoring with improved consistency; the current trend is to incorporate automated TILs as complementary variables within broader prognostic and predictive models rather than completely replacing the visual assessment [151,152].

While conventional IHC platforms assess a few markers per slide, multiplex IHC and multiplex immunofluorescence (mIF) platforms enable simultaneous analysis of multiple antigens while preserving spatial information.

Technologies such as sequential chromogenic multiplexing, multicycle mIF, imaging mass cytometry, MIBI, CycIF, and digital spatial profiling [153,154] enable the following:

- Detailed characterization of the immune microenvironment (T-cell subsets, B cells, macrophages, dendritic cells, etc.) in breast tumors;
- Identification of spatial inflammatory phenotypes, such as “hot” tumors, “immune-excluded” tumors (lymphocytes restricted to peripheral stroma), and “immune desert” patterns;
- Measurement of the distances and interactions between tumor and immune cells (e.g., the proximity of CD8+ lymphocytes to tumor cells), with potential prognostic and predictive value for neoadjuvant chemotherapy or immunotherapy response [152].

Conceptually, use of digital pathology and advanced IHC have shifted the role of pathologists from slide interpretation to the curation of morphologic and molecular data, bridging microscale phenomena (cells, proteins, spatial interactions) and macrolevel therapeutic decisions [153].

These innovations, combined with AI and large-scale analytics, are transforming contemporary oncology [154,155]. The future of breast cancer management is moving toward multiplatform, adaptive, and personalized approaches in which clinical algorithms integrate genomic, immunologic, and functional data to guide treatment in real time [156].

## 3. Advances in Breast Cancer Research and Perspectives: Where Are We Going?

Breast cancer management is transitioning from predominantly morphology- and receptor-based decision-making to an integrated precision paradigm in which molecular profiling, immune characterization, and computational tools increasingly influence both prognostic assessment and therapeutic selection. Importantly, the translational significance of these advances is not determined solely by biological plausibility or technical sophistication, but by their ability to generate reproducible clinical benefit, to be implemented in routine workflows, and to remain equitable across diverse health systems.

A central theme emerging from contemporary evidence is that therapeutic innovation has advanced at different “maturity levels”. The most practice-changing gains to date have been driven by modalities that are scalable and deployable across broad patient populations, most notably antibody–drug conjugates (ADCs) and immune checkpoint inhibitors (ICIs). ADCs have been particularly disruptive because they can partly mitigate antigen heterogeneity through high-potency payloads and, in some constructs, bystander effects, thereby extending targeted delivery beyond classical oncogene-addicted phenotypes. Conversely, the clinical benefit of ICIs in breast cancer has proven highly context dependent, with more consistent efficacy in TNBC and stronger signals when introduced earlier in the disease course and/or under biomarker-enriched conditions. These observations collectively reinforce a mechanistic principle with practical implications: modalities capable of overcoming tumor heterogeneity and immune escape tend to produce more reproducible time-to-event gains, provided that patient selection and treatment context are aligned with the underlying biology.

In parallel, diagnostic innovation is shifting from “static classification” to “longitudinal inference”. Liquid biopsy exemplifies this transition by enabling the dynamic tracking of clonal evolution and resistance mechanisms. Nevertheless, broad adoption requires careful distinction between prognostic and predictive utility. While ctDNA detection may identify molecular relapse or emergent resistance earlier than imaging, clinical benefit depends on whether acting on these signals improves patient-centered outcomes and survival rather than merely advancing the time of detection. Thus, prospective intervention-triggered trials and assay harmonization remain necessary before ctDNA-guided escalation/de-escalation strategies can be widely implemented outside selected scenarios.

Functional precision platforms, such as patient-derived organoids and organoid-on-a-chip systems, offer a conceptually attractive complement to genomics by capturing emergent phenotypes not reducible to single alterations. However, these approaches remain constrained by standardization challenges, turnaround time, and uncertain generalizability across tumor subclones and microenvironmental contexts. Their near-term clinical value may be greatest in tightly defined use cases (e.g., prioritization among plausible therapies in heavily pretreated disease) and when integrated with molecular data rather than positioned as standalone arbiters of treatment selection. Similarly, cellular engineering and bispecific immune redirection represent the leading edge of immune engineering, but the gap between feasibility and durable efficacy in solid tumors remains substantial, largely due to trafficking barriers, antigen heterogeneity, immunosuppressive microenvironments, and scalability limitations.

Collectively, these lines of evidence argue that the next phase of precision breast oncology will be determined by integration rather than incremental expansion of any single technology. High impact progress is most likely where diagnostic frameworks converge with rational therapeutic sequencing: deploying biomarkers that are not only measurable but actionable; selecting therapies that match tumor vulnerability and immune context; and anticipating resistance with longitudinal monitoring strategies. This also highlights the growing importance of implementation science in oncology: reproducibility, workflow integration, toxicity management, cost-effectiveness, and regulatory validation increasingly shape real-world impact as much as mechanistic innovation.

Despite major advances, several limitations constrain the translation of emerging technologies into routine breast cancer care. First, heterogeneity in biomarker assays (e.g., PD-L1 scoring platforms, HER2-low definitions, ctDNA analytical pipelines) limits cross-study comparability and may affect reproducibility in real-world settings. Second, evidence supporting longitudinal strategies (notably ctDNA-guided escalation/de-escalation) remains incomplete without prospective, intervention-triggered trials demonstrating improved survival or patient-centered outcomes beyond earlier detection. Third, functional precision platforms (organoids/PDOs) and immune engineering approaches (CAR-T, bispecific constructs) require further standardization, scalability, and validation in clinically meaningful endpoints for solid tumors. Fourth, toxicity management and optimal sequencing, particularly across ADC payload classes and immunotherapy combinations, remain unresolved and demand biomarker-informed algorithms.

Finally, future research should prioritize integrative, multimodal models that connect digital pathology, multi-omics, and immune profiling with pragmatic implementation strategies to reduce global disparities and to enable scalable precision oncology across diverse health systems, while also considering that, notwithstanding the growing adoption of artificial intelligence (AI) in clinical practice, several limitations remain. The heterogeneity and lack of standardization of molecular and clinical datasets restrict model robustness and generalizability across different populations and platforms, while nonrepresentative training data may introduce algorithmic bias. In addition, the limited interpretability of complex AI models hampers biological insight and clinical trust, particularly in molecular oncology, where batch effects and platform-specific variability affect reproducibility. The absence of large-scale prospective validation and seamless integration into clinical workflows further constrains the translational impact of AI, highlighting the need for rigorous validation and standardized implementation frameworks to ensure safe and effective use in cancer care [157,158].

A major and often under discussed implication of this rapidly evolving landscape is the disparity between high-income settings and low- and middle-income countries (LMICs). Many tools emphasized in precision oncology comprehensive NGS, multigene assays, ctDNA MRD programs, and digital pathology/AI infrastructure are concentrated in well-resourced centers, whereas substantial fractions of the global breast cancer burden occur in settings where access to timely diagnosis, quality-assured ER/PR/HER2 testing, radiotherapy capacity, consistent drug supply, and supportive care remains limited [159]. This creates a two-tier reality: in high-income systems, incremental outcome gains are increasingly pursued through biomarker refinement and next-generation therapeutics; in resource-constrained systems, avoidable mortality may still be driven by late-stage presentation, fragmented referral pathways, and incomplete access to established standard-of-care treatments.

From a translational perspective, this disparity matters because it can distort the perceived “global” impact of innovation. The greatest population level gains in many LMIC contexts may still derive from strengthening foundational oncology services, such as organized screening and early detection, robust pathology with quality control, timely surgery and radiotherapy, and uninterrupted access to essential systemic therapies, while adopting precision technologies through a tiered, high-yield strategy. Pragmatically, prioritizing diagnostics and therapies that directly alter management (e.g., validated receptor testing, focused mutation testing linked to available drugs, and scalable digital pathology for teleconsultation and standardized reporting) may generate more immediate benefit than attempting to replicate high-cost, high-complexity precision pipelines without the necessary infrastructure.

Nevertheless, structural constraints should not justify the exclusion of low- and middle-income countries (LMICs) from technological innovation in breast cancer care. On the contrary, equitable implementation strategies are required to ensure that advances in diagnostics, digital pathology, and systemic therapies are adapted to diverse healthcare settings. Scalable, cost-effective, and context-aware innovations are essential to prevent the widening of global disparities and to enable meaningful participation of LMICs in the evolving landscape of precision oncology.

Accordingly, future research and policy should pursue dual objectives: (i) continued development of biomarker-driven therapeutics and longitudinal diagnostics that improve outcomes, and (ii) implementation strategies that ensure innovations translate into real-world survival gains across diverse health systems, including trial designs that include underrepresented regions and pragmatic evaluations of feasibility, cost, and clinical utility.

Thus, the next step in precision oncology for treating breast cancer is not only to develop new technologies but to ensure that they are clinically meaningful, cost effective, and implementable in routine patient care.

## 4. Conclusions

Breast cancer care is rapidly transitioning toward precision oncology, driven by integrated molecular and immune profiling, advances in digital and computational pathology, and the development of next-generation therapeutics. Emerging technologies, including artificial intelligence, machine learning, data mining, and wearable devices, are enhancing tumor characterization, supporting drug development, and enabling more personalized and data-driven clinical decision-making. However, the principal challenge remains translating these innovations into clinically meaningful, scalable, and equitable benefits. Future progress will depend on integrating diagnostics and therapeutics into actionable clinical frameworks while ensuring cost-effectiveness and accessibility across diverse healthcare systems, so that technological advances result in improved survival and quality-of-life outcomes worldwide. Furthermore, physicians must enhance their understanding of available resources, critically interpret results, and rationally integrate them into decision algorithms, ensuring that technological advances translate into improved survival, reduced toxicity, and better alignment with each patient’s preferences and values.

## Figures and Tables

**Table 1:** Immunological, Targeted, and Antibody–Drug Conjugate Strategies in Breast Cancer.

Strategy	Trial (Phase)	Setting/Line	Agent(s)	*n*	Primary Endpoint	Main Outcome	Ref.
Tumor microenvironment targeting	E2100 (III)	1L mBC (HER2−)	Paclitaxel + bevacizumab vs. paclitaxel	722	PFS	↑ Median PFS 11.8 vs. 5.9 mo.	[30]
Immune biomarkers (TILs)	Pooled analysis	Early BC (all)	NA	>12,000	DFS, OS	High stromal TILs associated with improved DFS/OS; strongest in TNBC/HER2+	[31]
Checkpoint inhibitors (CTLA-4)	Tremelimumab + exemestane (I)	Metastatic HR+/HER2−	Tremelimumab + exemestane	26	Safety/immune	No ORR; immune activation observed	[32]
Checkpoint inhibitors (CTLA-4)	Ipilimumab + cryoablation (I)	Early BC (window)	Ipilimumab post-cryoablation	19	Immune activation	Increased TILs and T-cell clonality	[33]
Checkpoint inhibitors (CTLA-4 + PD-1)	Ipilimumab + nivolumab (I/II)	Advanced TNBC	Ipilimumab + nivolumab	38	ORR, safety	Modest ORR	[34]
Checkpoint inhibitors (CTLA-4 + PD-1)	Combination anti-CTLA-4/PD-1 (Phase I/II)	Adv solid tumors incl. TNBC	Ipilimumab + nivolumab vs. nivolumab	Subgroup	Safety, efficacy	Limited incremental benefit in TNBC	[35]
Checkpoint inhibitors	KEYNOTE-522 (III)	Stage II–III TNBC (neo + adj)	Pembrolizumab + CT vs. CT	1174	pCR, EFS	5 y OS 86.6% vs. 81.7%; *p* = 0.002	[36]
Checkpoint inhibitors	KEYNOTE-355 (III)	1L mTNBC	Pembrolizumab + CT vs. placebo + CT	847	PFS, OS	PD-L1 CPS ≥ 10: ↑ PFS 9.7 vs. 5.6 mo; OS 23.0 vs. 16.1 mo	[37]
Checkpoint inhibitors	IMpassion130 (III)	1L mTNBC	Atezolizumab + nab-paclitaxel	902	PFS, OS	OS benefit confined to PD-L1+ subgroup	[38,39]
Checkpoint inhibitors	GeparNuevo (II)	Early TNBC (neo)	Durvalumab + NACT vs. Placebo + NACT	174	pCR	No pCR gain; ↑ long-term iDFS/OS	[40]
Checkpoint inhibitors	TORCHLIGHT (III)	1L mTNBC	Toripalimab + nab-paclitaxel vs. Placebo + nab-paclitaxel	531	PFS	PD-L1+: PFS 8.4 vs. 5.6 mo; OS 32.8 vs. 19.5 mo	[41]
CAR-T	cMET RNA-CAR-T (I)	mTNBC/melanoma	RNA CAR-T (no lymphodepletion)	7 (4mTNBC)	Safety	No ≥ G3 toxicity; best response SD (4)/PD (3)	[42]
Vaccine	PRESENT/NeuVax (III)	Adj HER2-low node-positive	Nelipepimut-S + GM-CSF vs. placebo	758	DFS	No DFS benefit; trial stopped for futility	[43]
DC vaccine	Autologous DC vaccine (I)	Adv/m	HER2-pulsed DC vaccine	54	Safety	Safe; immune responses; disease stabilization	[44]
Bispecific antibody	KN026 (I)	Adv/m HER2+	KN026	57	Safety	Manageable toxicity; preliminary responses	[45]
Bispecific antibody	Ertumaxomab (I)	Metastatic BC	Trifunctional anti-HER2 anti-CD3	17 (15 evaluable)	Safety	Responses in 5/15 evaluable patients	[46]
ADC (HER2+)	EMILIA (III)	Adv/m HER2+ BC after prior trastuzumab and taxane	T-DM1 vs. LC	991	PFS, OS	↑ PFS 9.6 vs. 6.4; OS 30.9 vs. 25.1 mo	[47]
ADC (HER2+)	DESTINY-Breast03 (III)	HER2+ mBC (2L)	T-DXd vs. T-DM1	524	PFS	↑ PFS 28.8 vs. 6.8 mo; HR 0.33	[48]
ADC (HER2-low)	DESTINY-Breast04 (III)	HER2-low mBC (2/3L)	T-DXd vs. TPC CT	557	PFS	↑ PFS 9.9 vs. 5.1 mo; ↑ OS 23.4 vs. 16.8 mo	[49]
ADC (HER2-low/ultralow)	DESTINY-Breast06 (III)	HR + HER2-low/ultralow mBC after ≥1 prior endocrine-based; no prior chemo for mBC	T-DXd vs. TPC CT	866	PFS	HER2-low ↑ PFS med 13.2 vs. 8.1 mo; ultralow HR 0.78 (med 13.2 vs. 8.3 mo)	[50]
ADCs (HER2+)	DESTINY-Breast09 (Phase III)	1L Adv/m HER2 + BC	T-DXd +P vs. THP	770	PFS	↑ Med PFS 40.7 vs. 26.9 mo	[51]
ADC (HER2+)	DESTINY-Breast05 (III)	High-risk HER2 + early/primary BC + res inv dis post-neoadjuvant taxane + anti-HER2	Adj T-DXd vs. T-DM1 for 14 cycles	1635	iDFS	iDFS events reduction 51 (6.2%) vs. 102 (12.5%)	[52]
ADC (HER2+)	DESTINY-Breast12 (IIIb/IV)	HER2+ a/mBC ± BMs	T-DXd (single-arm, open-label)	504	PFS, ORR	BMs: 12 mo PFS 61.6%; CNS ORR 71.7%. OS immature	[53]
ADC (HER2+)	TULIP (III)	HER2+ mBC after ≥2 HER2 therapies/post-T-DM1	T-Duo (SYD985) vs. TPC CT	437	PFS	↑ PFS 7.0 vs. 4.9 mo	[54]
ADC (HER2+)	ACE-Breast-02 (III)	HER2+ mBC post-trastuzumab	ARX788 vs. LC	441	PFS	↑ PFS 11.3 vs. 8.2 mo	[55]
ADC (HER3)	TUXEDO-3 (II)	LMD/BM (solid tumors)	Patritumab-DXd	20	3-mo OS (proportion alive)	65% alive at 3 mo	[56]
ADC (TROP2)	ASCENT-03 (III)	mTNBC ≥ 2L	SG vs. TPC CT	529	PFS	PFS 4.8 vs. 1.7 mo; OS 11.8 vs. 6.9 mo	[57]
ADC (TROP2)	ASCENT-04 (III)	1L PD-L1+ mTNBC	SG + pembro vs. TPC CT + pembro	443	PFS	Med PFS 11.2 vs. 7.8 mo; OS 11.8 vs. 6.9 mo	[58]
ADC (TROP2)	TROPiCS-02 (III)	HR+/HER2− heavily pretreatment mBC	SG vs. TPC CT	543	OS	↑ OS 14.4 vs. 11.2 mo; HR 0.79	[57]
ADC (TROP2)	TROPION-Breast01 (III)	HR+/HER2− mBC progression on 1–2 prior chemo in inoperable/m setting)	Dato-DXd vs. TPC CT	732	PFS, OS	Final OS HR 1.01 (NS)	[59]

Abbreviations: ↑, increase; 1L, first-line; 2L, second-line; ≥2L, second line or later; BC, breast cancer; CAR-T, chimeric antigen receptor T-cell; CPS, combined positive score; DFS, disease-free survival; EFS, event-free survival; GM-CSF, granulocyte–macrophage colony-stimulating factor; HER2, human epidermal growth factor receptor 2; HR, hazard ratio; HR+, hormone receptor–positive; iDFS, invasive disease-free survival; mBC, metastatic breast cancer; mTNBC, metastatic triple-negative breast cancer; mo, months; y, years; *n*, number of patients; NACT, neoadjuvant chemotherapy; NS, not significant; OS, overall survival; pCR, pathologic complete response; PD, progressive disease; PD-1, programmed cell death protein 1; PD-L1, programmed death-ligand 1; PFS, progression-free survival; SD, stable disease; TNBC, triple-negative breast cancer; TPC, treatment of physician’s choice; T-DM1, trastuzumab emtansine; T-DXd, trastuzumab deruxtecan; vs., versus.

## Data Availability

Data sharing is not applicable to this article as no new data were created or analyzed in this study.
